# Supplementary material for: rtestim: Time-varying reproduction number estimation with trend filtering
Source: PLoS Comput Biol. 2024 Aug 6;20(8):e1012324. doi: 10.1371/journal.pcbi.1012324 (PMC11329163; doi:10.1371/journal.pcbi.1012324)
Supplement: Attachment — Submitted filename: response_to_reviewers.pdf [file pcbi.1012324.s004.pdf]

Response to the reviewers

We thank the reviewers for their careful and thoughtful assessment of our work and for raising a number of key points which will undoubtedly improve the manuscript.

Here we highlight some of the major changes we have made to the manuscript. The first is to add a number of experiments to compare the performance of our method with alternatives under several scenarios, following suggestions by the reviewers:

1. investigating misspecified serial interval distributions (under mild and more major misspecification);
2. including all degrees (k) of the discrete splines in our method across all settings, (thus investigating potential mismatches);
3. using relatively lower incident cases (< 1000) in addition to the previous settings;
4. examining epidemics that are shorter (with 50 timepoints); and
5. directly investigating interval coverage.

All experiments are now based on serial interval distributions for real epidemics (rather than synthetic distributions). We have included experimental results from EpiFilter and EpiNow2 wherever possible.

Another key change is that we clarify that the "instantaneous reproduction number" is our focus. With this in mind, we have modified the title to misstating our intentions and also to be aligned more closely with existing related work, e.g., [1, 5, 10, 13]. We also add more discussion on how the hyperparameters (degree k and tuning parameter λ) should be chosen in practice, the coverage of the approximate confidence interval, and a few future directions. Other changes include using time-varying serial interval distributions for Covid-19 incidence in Canada, discussing the level of overdispersion in the experiments, applying alternative methods to real epidemics for comparison to ours, wording clarification, and additional references.

In the following we address their concerns point by point. We first copy each reviewer's point and place our response directly below it, indented, using a slightly different typeface, and in blue.

Reviewer 1

Reviewer Point P 1.1 — There should be more citations in the first paragraph of the introduction.

> **Reply**: We've added more references to support our statements on the definition, importance, and interpretation of effective and instantaneous reproduction numbers and give examples on the $\mathcal{R}_t$ procedures we mentioned in the first paragraph.

Reviewer Point P 1.2 — It is unclear why the serial interval parameterizations are being changed for each simulation scenario and how this impacts the results. If these are shape and scale parameters, then this would imply really large means and variances (e.g. mean=$3.5 * 3.5 = 12.25$ and variance=3.5^3). It is not clear how these distributions are motivated.

> **Reply**: Previously, we had chosen these arbitrarily. Now, we have pivoted all simulations to use the estimated mean and standard deviations for real epidemics (specifically, flu, measles and

SARS) in existing literature [3, 7, 11, 12]. (Using estimated delays from real epidemics for the simulation experiments is inspired by [5].)

Reviewer Point P 1.3 — The negative binomial simulations chose a dispersion parameter that is relatively large and it is not clear that is a really strong violation of the Poisson assumption (unless some other parametrization of the distribution is being used).

Reply: We should have been explicit in describing our parameterization. The one we use leads to large overdispersion.

We use a parametrization with mean $\mu(t)$ and dispersion ρ. The probability mass function is then written as

$$p(y_t \mid \mu(t), \rho) = \frac{\Gamma(y_t + \rho)}{\Gamma(\rho) y_t!} \Big(\frac{\rho}{\rho + \mu(t)}\Big)^{\rho} \Big(\frac{\mu(t)}{\rho + \mu(t)}\Big)^{y_t},$$

where the probability of success is $p(t) = \rho/(\rho + \mu(t))$ and the variance is $\sigma(t)^2 = \mu(t)(1 + \mu(t)/\rho)$. (This parameterization is the same as in, e.g., [10]). Therefore, for any $\rho > 0$, this results in overdispersion relative to the Poisson distribution, and small values of ρ result in greater overdispersion. We added this context in lines 337–341 in Section 3.1.1 of the manuscript and also added a figure to the Supplement (Figure A.1.1) displaying the ratio of the variance divided by the mean for a simulated epidemic in each experimental setting, illustrating how much overdispersion is typical.

Reviewer Point P 1.4 — It would be helpful to see the method compared to EpiFilter in a setting where the case counts are smaller and EpiFilter would work. These scenarios are frequently of interest.

Reply: We have restructured our experiments to compare EpiFilter with our RtEstim and other alternatives using synthetic epidemics with relatively small incidence (≤ 1000) and large incidence (1K–10K) as well. An example of each setting for each epidemic is provided in Fig 2 in the manuscript. Details on the new experimental settings and additional results are added in the Results section (see Fig 3 for low incidence and Fig 4 for large incidence) in the manuscript and in Section A.3 (see the top two rows in Figures A.3.1 and A.3.2 for low incidence and the bottom two rows for large incidence) in the Supplement.

Reviewer Point P 1.5 — The choice of k varies for each scenario—how is one to decide what to use?

Reply: Previously, we had chosen k to match the structure of the true curve. This is a bit unfair. We have now used $k = 0, 1, 2, 3$ in all scenarios. There are some important points here (discussion of which we have also added to lines 398–407 in Section 3.1.2 in the manuscript):

- When faced with real data, the choice of k should be done either (1) based on the analyst's preference for the result (e.g., "I want to find large jumps, so $k = 0$") or (2) in a data-driven manner, as a component of the estimation process. While the manuscript previously focused on the first case, our software enables the second case as well: simply fit for different k and choose the pair k, λ that has smallest CV score. Thus, all necessary choices can be accomplished based solely on the data.

- Our software is a departure from existing methods in that we *allow* this choice and provide simple data-driven methods to accomplish it. EpiEstim has no such facility (implicitly, one must somehow choose the size of the rolling window, though its connection to the function class of the result is unclear). EpiFilter effectively uses cubic splines (similar to $k = 3$, but simply continuous rather than piecewise continuous). EpiLPS directly uses the cubic B-spline basis coupled with a gamma prior, which is also similar $k = 3$. EpiNow2 allows various choices through Gaussian Process kernels, and while one can put priors on the parameters of the kernel, the choice of kernel is required. None of these come with methods for evaluating these choices.
- In our experience, using $k > 3$ is nearly indistinguishable from $k = 3$, though it is allowed. So if the analyst somehow imagines "$\mathcal{R}_t$ is best described by a 10th order piecewise polynomial" then the software can easily accommodate this desire.

Reviewer Point P 1.6 — It would be helpful to see how sensitive the simulation results are to the assumed serial interval distribution. Presumably some methods should be more sensitive to this than others.

> **Reply**: This is a great idea. We have added experiments to compare the accuracy of different methods by using misspecified serial interval (SI) distributions. We consider two cases, mild and major misspecification. To create these scenarios, we use the estimated SI distributions from real epidemics (measles, SARS and flu) to generate the synthetic date. In "mild" misspecification of a SI distribution, at estimation time, we increase the mean by 2 and the standard deviation by 10%, while in "major" misspecification, we use the SI distribution from a different disease for estimation than was used in simulation (swapping SARS for measles, say). The densities for each SI distribution are displayed in Figure A.1.2 for reference. The related experimental results are provided in Section A.4 in the Supplement.

Reviewer Point P 1.7 — It would be helpful to compare RtEstim with EpiEstim and other approaches for the real-world examples, too. This would provide insight on whether the patterns observed in the simulations hold up with real data.

> **Reply**: We have added this in Section A.8 in the Supplement. See Figures A.8.1 and A.8.2 for Covid-19 cases in Canada and Figures A.8.3 and A.8.4 for influenza in Baltimore, Maryland in 1918.

Reviewer Point P 1.8 — I do not really follow the discussion on the reporting delay being inconsequential.

> **Reply**: We suspect the reviewer is referring to line 142 in the (original) manuscript. We did not intend to give the impression with this discussion that reporting delay is inconsequential. But rather to argue, that under certain conditions, failing to account for the reporting delay will minimally impact the accuracy of any $\mathcal{R}_t$ estimator that is based on Eq. (2). We have tried to clarify the language there in lines 169–177 in the (revised) manuscript.

Minor

Reviewer Point P 1.9 — l2-3. The instantaneous reproductive number is a type of effective reproductive number. These are not interchangeable.

> **Reply**: Thank you for pointing this out. We've clarified the language in lines 6–8 by defining the instantenous reproduction number and we only focus on estimating the instanteneous reproduction number in the rest of the manuscript.

Reviewer 2

Reviewer Point P 2.1 — Before I felt comfortable using this estimator, I would want to see additional analyses examining robustness to GI misspecification. Is this automated smoothing procedure robust to misspecified GIs? Could it potentially compound GI misspecification, adding additional bias? On the other hand, it seems possible that the smoothing induced by the penalty might could reduce the bias from GI misspecification. I'd be curious to see the impact on the simulated situations in Figure 2.

> **Reply**: This is also a point made by Reviewer 1 (P 1.6). We have added experiments to compare the accuracy of different methods by using misspecified interval distributions. We consider two cases: mild and major misspecification. To create these scenarios, we use the estimated SI distributions from real epidemics (measles, SARS and flu) to generate the synthetic date. In "mild" misspecification of a SI distribution, at estimation time, we increase the mean by 2 and the standard deviation by 10%, while in "major" misspecification, we use the SI distribution from a different disease for estimation than was used in simulation (swapping SARS for measles, say). The densities for each SI distribution are displayed in Figure A.1.2 for reference. The related experimental results are provided in Section A.4 in the Supplement.
>
> The experimental results support that (1) our method is reasonably robust to the misspecification overall, and (2) in comparison to other methods under the same experimental settings, our method performs at least as well as the best of the alternatives (with lowest KL divergence).

Reviewer Point P 2.2 — Do the confidence intervals cover Rt at the nominal levels? Is this true in the negative binomial case? What about for predicted incident cases?

> **Reply**: This is an important point, but complicated. We have done some evaluation to address it, but despite "good" performance, we would hesitate to attach much significance to the results. We will first explain the reasons for our hesitation before describing the set of experiments undertaken to investigate the reviewer's questions. We also added a feature to provide the CIs for the predicted incident cases. However, we have not evaluated it in simulations, since it's not a main focus of the paper, and given the additional experiments already included. In real applications, it is not clear what to gain from these CIs. Essentially, these say "if another epidemic occurred over this time with the same data generating process, then we would expect

cases to fall in this band." They don't address systematic or random measurement errors in the data, which would be desirable.

Accurate statistical coverage of a function is a difficult problem, and the types of (frequentist) guarantees that can be made are not always what one would want [8]. So, theoretically, whether these intervals should be expected to provide $(1-\alpha)\%$ coverage simultaneously over all time while being narrow enough to provide useful uncertainty quantification is neither easy nor settled. Empirically, our observations for our method, as well as all others we have seen, follow a similar (undesirable) pattern: when $\mathcal{R}_t$ is stable, they *over cover* dramatically (even implausibly narrow intervals have 100% coverage); but when $\mathcal{R}_t$ changes abruptly, they *under cover* (coverage drops to nearly 0%). Taken together, since the abrupt changes are fairly rare in the simulation settings, coverage appears close to nominal (our method and others achieve approximately 95% coverage on average). But, ideally, public health would much prefer accurate coverage in periods when $\mathcal{R}_t$ is rapidly changing, precisely when these methods are the worst. Furthermore, the shapes of the intervals are not believable: they should be asymmetric, getting wide below the estimates preceding a decrease in $\mathcal{R}_t$ (and the opposite for an increase). No method that we've seen produces these shapes. We would suggest that this is a major open problem in the field, and likely one without easy answers. We note that similar coverage issues plagued forecasting performance during the pandemic [16, 6].

Experimental settings and results.

We added a set of experiments to evaluate the coverage level of 95% confidence intervals (CIs) of our method and other alternatives on Poisson and Negative Binomial incident cases. We consider three metrics:

1. we display the average coverage (over 50 random epidemics) per time point for all methods under each experimental setting,
2. we compute the percent coverage averaged across all time and random realizations per method per setting,
3. and we compute the interval score (IS) (e.g. [4]). It is defined as

$$\mathrm{IS}_\alpha(\mathcal{R}, u, l) = \frac{1}{n}\sum_{t=1}^{n}(u_t - l_t) + \frac{2}{\alpha}(l_t - \mathcal{R}_t)\mathbf{1}(\mathcal{R}_t < l_t) + \frac{2}{\alpha}(\mathcal{R}_t - u_t)\mathbf{1}(\mathcal{R}_t > u_t),$$

   where $\alpha = 0.05$ is the significance level, l, u are the lower and upper bounds of the confidence band, $\mathcal{R}$ is the true effective reproduction number, and $\mathbf{1}(A) = 1$ if event A occurs and 0 otherwise. A confidence band that covers the true value more frequently with a shorter interval width will have a lower interval score.

We found that (in Figures A.6.1–A.6.4, illustration of sample epidemics) our confidence bands are generally much wider than competitors, especially at the early stage. They tend to over cover, whereas others tend to under cover. We also found that (in Figures A.6.5 and A.6.6, coverage across time) low Poisson incidence is the easiest for all methods, with coverage near 100% at most timepoints and 0 elsewhere. Large negative Binomial incidence is the hardest, though EpiLPS does the best here. RtEstim with $k = 0$ tends to produce overly narrow intervals, leading to lower coverage. In Figures A.6.7 and A.6.8 (averaged coverage), CIs of RtEstim with $k = 1, 2, 3$ have nearly 100% coverage across all timepoints for all random samples except in the hardest problem, where the incidence is large and overdispersed. The coverage of RtEstim $k = 0$

is lower than for other degrees, similar to the above. RtEstim (in Figures A.6.9 and A.6.10) always has the lowest or close to the lowest interval scores. In Poisson cases, EpiFilter has the lowest interval scores (less than 1), and the scores of RtEstim are around 1.

Reviewer Point P 2.3 — In lines 65–66, the authors describe this estimator as a "retrospective effective reproductive number". I'm confused by this phrase. Does it mean that the proposed method is not expected to forecast well and should only be used retrospectively? Or does it mean that the estimator is a case reproductive number rather than an instantaneous reproductive number [9]? If it is a case reproductive number, differences with the Cori method's instantaneous reproductive number should be discussed.

Reply: We should have been more clear. Our meaning of "retrospective" is that we estimate the instantaneous reproduction number at all times t given the entire sequence of observed incidence of length n for $t = 1, \ldots, t, \ldots, n$. Currently, our method does not forecast beyond the latest time n mainly due to the difficulty of extrapolation in nonparametric regression. We are currently exploring and evaluating methods to overcome this challenge.

Reviewer Point P 2.4 — For the comparisons, why use EpiEstim vs. the newer and more robust EpiNow2? The GP in EpiNow2 also provides smoothing, so it should be a stronger comparison.

Reply: The main reason that we had excluded EpiNow2 in our original experiments is that it is very slow, whereas our method, EpiEstim, EpiFilter, and EpiLPS, are all orders of magnitude faster. It also does much more than these methods, so it's not really an apples to apples comparison.

We've now added experiments with shorter epidemics (see Section A.3.2 in the Supplement) that allow for some comparisons. In these situations, as the reviewer mentions, EpiNow2 is a strong competitor and can be as accurate as our method under multiple settings and win in some cases.

Reviewer Point P 2.5 — I'm not sure the timeseries of Rt in Figures 1 & 7 are appropriate because of the change in the GI during the several years displayed. I would think the bias would be particularly severe during the Omicron wave, when the model is at its most certain. It would probably be more appropriate to use different GIs for before and after Omicron's emergence (with different model fits perhaps).

Reply: We did a few things to address this point. We first added a feature in the software to allow the input of different delays for different timepoints. Now, different (non-parametric) delays can be used at each time t if desired (previously, we allowed time-constant non-parametric delays.) Second, we redid the analysis displayed in Fig 1 in the manuscript, specifying different delays by dominant variant. We discuss the specific choices of delay parameters more carefully in the manuscript and the Supplement. However, in the comparisons with other alternatives, we stick with a single delay, since other methods don't generally allow time-varying delays.

Minor

Reviewer Point P 2.6 — I had some trouble following the notation in line 184. Does the $D^{(1)}$ case correspond to $k = 0$? If so, it would be helpful to say that explicitly.

> **Reply**: Yes, $D^{(1)}$ is the divided difference matrix for trend filtering penalty with $k = 0$ with dimension $(n-1) \times n$. We've clarified it in lines 212–213 in the manuscript.

Reviewer Point P 2.7 — In Figures 4 & 5, I had trouble distinguishing the colors in all the different situations.

> **Reply**: We added a figure to display each estimated Rt curve in separate panels in Figures A.4.1–A.4.4 in the Supplement for the same set of examples with Poisson and negative binomial incidence resepctively.

Reviewer Point P 2.8 — In Figures 4 & 5, does the RtEstim (k=0) case appear in all the panels?

> **Reply**: It was only used for the first scenario (piecewise constant) in our original experiments. We've now applied it to all scenarios.

Reviewer Point P 2.9 — I don't believe the norm used in section 2.3 is ever defined. Is it the L1 norm?

> **Reply**: We used the ℓ_1 norm and the infinity norm in Section 2.3. We've added the definition of ℓ_1 norm in line 210 in Section 2.2 when it's first mentioned and the definition of the infinity norm in lines 258–259 in Section 2.3 in the manuscript.

Reviewer Point P 2.10 — I would appreciate seeing the values plotted in Figures 4 & 5 as the difference between the fitted and true values, perhaps as additional supplementary figures. It's nice to see the timeseries, but I have trouble reading the figures.

> **Reply**: Since we modified the figures in the manuscript, the relevant figures are now labelled as Fig 5 and Fig 6. We added the relevant full visualization in Figures A.6.1 and A.6.4 in the Supplement.

Reviewer Point P 2.11 — In Section 2.4, I would appreciate some discussion of why random splitting into k folds is more appropriate than leave future out validation.

> **Reply**: Our method doesn't forecast (as mentioned above), so this isn't an option. We allow for either random or regular splitting (where we assign every k^{th} observation into the same fold). Our approach is most closely related to non-parametric regression rather than time series forecasting. That said, under some conditions, one can guarantee that K-fold is valid for risk estimation in time series. The sufficient conditions are somewhat strong, but the guarantees are also stronger than would be required for model selection consistency [2].

Reviewer Point P 2.12 — I do not think the claims on line 430–440 in the Discussion around epidemic forecasting and real-time usage are appropriate or substantiated by the analysis. The authors do not evaluate performance at forecasting Rt and, without seeing an evaluation, I am skeptical that splines would outperform other methods.

> **Reply**: This is a good point. We have removed it.

Reviewer Point P 2.13 — I would appreciate some more discussion of outlier handling. In several places, it is mentioned that outliers are excluded but I couldn't find what qualified as an outlier or why.

> **Reply**: Sorry for the confusion. These were not about "outliers" in the data, only about outliers displayed on the boxplots. The purpose was be able to focus on the bulk of the simulation results. We don't display these in the figures in the main manuscript because a few extremely large values dominate the y-axes, making the figures hard to read. We have added the full figures in Figure A.3.1 in the the Supplement. We had also clarified this in the captions of Figures 3 & 4 in the manuscript.

Reviewer Point P 2.14 — When discussing the generation interval, its approximation by the serial interval, and discretization procedures it would be good to discuss the results in [14] and [15].

> **Reply**: Good point. Added.

Reviewer 3

Reviewer Point P 3.1 — The results shown in Figures 3 and 4 are very promising, though I am wondering how sensitive the proposed method is to the choice of smoothness degree k of the discrete splines. In the context of the synthetic datasets (both Figure 3 and 10 and 11) it seems that the choice of the parameter k plays an important role in whether the RtEstim method will outperform the other two considered, as it can be observed by comparing the means in Scenario 2.

> **Reply**: This is a point also made by Reviewer 1 (P 1.5), and an important one that we should make more clear in the Manuscript. We recapitulate that discussion here for convenience. Hopefully this also addresses the reviewer's next point. Previously, we had chosen k to match the structure of the true curve. This is a bit unfair. We have now used $k = 0, 1, 2, 3$ in all scenarios. There are some important points here (discussion of which we have also added to the manuscript in lines 398–407 in Section 3.1.2):
>
> - When faced with real data, the choice of k should be done either (1) based on the analyst's preference for the result (e.g., "I want to find large jumps, so $k = 0$") or (2) in a data-driven manner, as a component of the estimation process. While the manuscript previously focused on the first case, our software enables the second case as well: simply fit for different k and choose the pair k, λ that has smallest CV score. Thus, all necessary choices can be accomplished based solely on the data.

- Our software is a departure from existing methods in that we *allow* this choice and provide simple data-driven methods to accomplish it. EpiEstim has no such facility (implicitly, one must somehow choose the size of the rolling window, though its connection to the function class of the result is unclear). EpiFilter effectively uses cubic splines (similar to $k = 3$, but simply continuous rather than piecewise continuous). EpiLPS directly uses the cubic B-spline basis coupled with a gamma prior, which is also similar $k = 3$. EpiNow2 allows various choices through Gaussian Process kernels, and while one can put priors on the parameters of the kernel, the choice of kernel is required. None of these come with methods for evaluating these choices.
- In our experience, using $k > 3$ is nearly indistinguishable from $k = 3$, though it is allowed. So if the analyst somehow imagines "$\mathcal{R}_t$ is best described by a 10th order piecewise polynomial" then the software can easily accommodate this desire.

Reviewer Point P 3.2 — Also, the EpiLPS seems to generate relatively similar results in the case of Scenario 2 to those produced by RtEstim, at a fraction of the time. Also, it is not very clear how well the proposed RtEstim method would behave in the context of a misspecified k parameter choice. This becomes really important in the real data analysis where we do not know a priori the type and number of temporal changes we can expect. A clearer explanation of the reason behind the choices of k made and how one would select an optimal k would be useful for completeness.

> **Reply**: Hopefully the previous point addresses most of this (and the enhanced discussion in the manuscript helps). We have noted the similarity of EpiLPS in Scenario 2 (and also performing well Scenario 4) in the manuscript. After revising the experiments in light of suggestions elsewhere, we find EpiLPS and RtEstim perform similarly (with good accuracy) in low incidence cases, and EpiLPS is better for large negative binomial incidence.

Minor

Reviewer Point P 3.3 — A more accurate definition of the effective reproduction number Rt is that it is the expected number of secondary infections produced by primary infection throughout the course of his entire infection if conditions remain the same as at time t.

> **Reply**: Thank you for pointing this out. This was also mentioned by another reviewer. We've modified the definition of effective reproduction numbers along these lines in lines 2–4 of the manuscript.

Reviewer Point P 3.4 — Also, in Line 220 using the phrase "M λ-values" would help improve clarity of text.

> **Reply**: We've added this explanation. It's now in line 250 in the manuscript.

Reviewer 4

Reviewer Point P 4.1 — p.13. EpiFilter would be an important comparator method here, as the authors have mentioned it several times earlier in their paper. However, they remove it from consideration after finding that it fails to converge for some of their synthetic data, due to the large incidence counts. However, I have actually been able to run EpiFilter to learn Rt successfully on incidence time series with larger numbers of daily cases than those considered in this paper. I appreciate that there may be some other features of the author's time series which cause EpiFilter to fail, but if EpiFilter is going to be discussed as a comparator earlier in the paper and then not actually used, I would value a more detailed explanation of why it cannot be applied in the setting considered by the authors.

> **Reply**: We've now revised the experiments to compare with EpiFilter. The issue was that default number of bins in the software was much too small. In the revision, we've increased these by an order of magnitude (matching the choices in the EpiFilter manuscript). Some experimental results are shown in the Result section (see Fig 3 and Fig 4) in the manuscript. Other results can be found in Section A.3 in the Supplement. Fig 5 and Fig 6 give examples for some experimental settings and others can be found in Section A.7.1 (see Figures A.7.1 and A.7.2) in the Supplement. Section A.6.1 in the Supplement gives a full visualization of $\mathcal{R}_t$ estimates an example of all methods for each setting.

Reviewer Point P 4.2 — p.14. These challenges with EpiFilter draw attention to another concern, which is that in their synthetic data experiments, despite considering a range of different shapes of true Rt profiles, the authors are always relying on time series which reach high daily incidence ($>$ 1000/day). It would be valuable to see how the methods perform when incidence is much lower.

> **Reply**: This point was also a concern to Reviewer 1. We have restructured our experiments with two separate conditions, adding synthetic epidemics with relatively small incidence (≤ 1000) to our settings which produce large incidence (1K–10K). An example of each setting for each epidemic is provided in Fig 2 in the manuscript. Details on the new experimental settings and additional results are added in the Result section (see Fig 3 for low incidence and Fig 4 for large incidence) in the manuscript and in Section A.3 (see the top two rows in Figures A.3.1 and A.3.2 for low incidence and the bottom two rows for large incidence) in the Supplement.
>
> An additional note here, because RtEstim directly optimizes the Poisson negative log-likelihood with the nonnegative mean on the log-scale, the size of the epidemic, theoretically, should not impact its results. On the other hand, EpiFilter optimizes by using something like a particle filter with a discretization over a range of possible $\mathcal{R}_t$. For this reason, larger incidence requires a larger number of grid cells inhibiting accuracy. While the software defaults cannot handle large epidemics, increasing the number of cells by a few orders of magnitude allows it to operate (though more slowly).

Reviewer Point P 4.3 — p.14. RtEstim computes Rt for a large grid of lambda values, with the best selected via cross validation. I acknowledge that tuning lambda is an integrated part of the RtEstim method, and the authors have developed an efficient way to perform this, whereas

hyperparameter tuning may be more of a computational burden with the other methods. However, at the very least there should be more discussion of how the tuning parameters of EpiEstim and EpiLPS were selected, and whether those values are appropriate for the problems at hand, given the possibility of a situation where the comparators are only underperforming due to inappropriate hyperparameter choices.

> **Reply**: This is an important point. As the reviewer mentions (and as described in responses regarding the choice of k above), for RtEstim, all tuning parameters can be chosen in a statistically rigorous, data driven way (using cross validation to estimate the expected error, and minimizing this estimate). Our goal in the manuscript was to emphasize this feature and compare the results to those that a typical user would achieve with alternative software. Therefore, we structured the simulations to "compare to best practices", and we used the software defaults or the choices that authors made in their original paper.
>
> Unfortunately, alternative software implementations do not provide similar procedures for choosing any necessary hyperparameters. This includes not just tuning parameters impacting the model, but also those for optimization (as mentioned above). For example, in EpiLPS, one must specify the number of basis functions as well as 5 prior parameters. EpiFilter needs a grid of possible R values, along with the a fixed value for the diffusion noise, and a prior value for $\mathcal{R}_1$. EpiNow2 has many prior parameters (depending on the particular model used) and does not directly compute a Bayes Factor or other model selection criterion. Had we attempted to tune some or all of these parameters, we would need to implement cross validation from scratch for each, as none of these provide ways to choose them. We felt that such an exercise is both beyond the scope of the current manuscript and unlikely to match the behaviour of a typical user. More likely, and likely to ill-effect, a typical user would look at the results and "turn some knobs" to produce a result that better matches their intuition.

Reviewer Point P 4.4 — p.17. EpiEstim and EpiLPS are both seeing drastic overestimation of Rt for the first few days of the time series. The authors mention this, but don't explain why it's happening. Is there something going wrong with the methods or their implementations to cause this? Are the results where RtEstim appears to outperform overall (Figure 3) arising just because the other methods have an overestimation error in the first few days?

> **Reply**: This happens for two reasons. First, some of these purposely impose strong priors larger than 1 to avoid over confidently asserting that an epidemic is under control. Second, the implementations suffer at the left boundary due to windowing behaviour. Our comparison ignores the first week of data to avoid making conclusions based solely on these details. One could argue that we should have left out more than a week, but one would hope for accurate estimates as early in the outbreak as possible. In some experiments, we see that this effect can persist for up to 30 days. We felt that ignoring 1 week is a good middle ground between requiring software to produce accurate estimates quickly (based on their own implementation) and removing the effects of minor artifacts at the beginning of the period that shouldn't be held against it.

Reviewer Point P 4.5 — p.23. If there is some substantial computational, theoretical, or implementation detail that makes imported cases difficult to incorporate into RtEstim, I am fine with the authors explaining this and deferring it to future work. But, would it actually be at all

difficult to allow the RtEstim method and software to incorporate imported cases, using a modelling framework such as [17]? Many of the comparator methods and software packages discussed in the paper already have the ability to handle imported cases.

Reply: This is a good idea that we hadn't considered. We don't forsee any difficulty incorporating this feature directly, using the framework of [17]. We have mentioned this as a possible extension in lines 556–559 in the section of Discussion.

Reviewer Point P 4.6 — General. I would argue that an important part of learning Rt is estimating the uncertainty in Rt implied by the data. In many situations, uncertainty in the value of Rt can be an important part of decision making. For example, if some measure of the best fit Rt for a particular incidence series falls below 1, but this is an uncertain estimate and there is still a decent chance that Rt is actually > 1, it would be highly unwise to conclude that the disease outbreak is currently under control. The authors develop confidence bounds for their method in section 2.5, but they don't make much use of them when they compare to other methods, nor do they discuss the implications of the confidence bounds when fitting to real data. Similarly, they discuss the robustness of their method and other methods to negative binomial stochasticity, but the focus again is on daily point estimates of Rt rather than considering to what extent the inferred uncertainty encompasses the true value of Rt.

Reply: This is an important point also mentioned by another reviewer (see P 2.2), but complicated. We consider three metrics:

1. we display the average coverage (over 50 random epidemics) per time point for all methods under each experimental setting,
2. we compute the percent coverage averaged across all time and random realizations per method per setting,
3. and we compute the interval score (IS) (e.g. [4]). It is defined as

$$\mathrm{IS}_\alpha(\mathcal{R}, u, l) = \frac{1}{n}\sum_{t=1}^{n}(u_t - l_t) + \frac{2}{\alpha}(l_t - \mathcal{R}_t)\mathbf{1}_{(\mathcal{R}_t < l_t)} + \frac{2}{\alpha}(\mathcal{R}_t - u_t)\mathbf{1}_{(\mathcal{R}_t > u_t)},$$

   where $\alpha = 0.05$ is the significance level, l, u are the lower and upper bounds of the confidence band, $\mathcal{R}$ is the true effective reproduction number, and $\mathbf{1}(A) = 1$ if event A occurs and 0 otherwise. A confidence band that covers the true value more frequently with a shorter interval width will have a lower interval score.

We found that (in Figures A.6.1–A.6.4, illustration of sample epidemics) our confidence bands are generally much wider than competitors, especially at the early stage. They tend to over cover, whereas others tend to under cover. We also found that (in Figures A.6.5 and A.6.6, coverage across time) low Poisson incidence is the easiest for all methods, with coverage near 100% at most timepoints and 0 elsewhere. Large negative Binomial incidence is the hardest, though EpiLPS does the best here. RtEstim with $k = 0$ tends to produce overly narrow intervals, leading to lower coverage. In Figures A.6.7 and A.6.8 (averaged coverage), CIs of RtEstim with $k = 1, 2, 3$ have nearly 100% coverage across all timepoints for all random samples except in the hardest problem, where the incidence is large and overdispersed. The coverage of RtEstim $k = 0$ is lower than for other degrees, similar to the above. RtEstim (in Figures A.6.9 and A.6.10)

always has the lowest or close to the lowest interval scores. In Poisson cases, EpiFilter has the lowest interval scores (less than 1), and the scores of RtEstim are around 1.

To expand on the reasons for mistrusting these intervals, accurate statistical coverage of a function is a difficult problem, and the types of (frequentist) guarantees that can be made are not always what one would want [8]. So, theoretically, whether these intervals should be expected to provide $(1-\alpha)\%$ coverage simultaneously over all time while being narrow enough to provide useful uncertainty quantification is neither easy nor settled. Empirically, our observations for our method, as well as all others we have seen, follow a similar (undesirable) pattern: when $\mathcal{R}_t$ is stable, they *over cover* dramatically (even implausibly narrow intervals have 100% coverage); but when $\mathcal{R}_t$ changes abruptly, they *under cover* (coverage drops to nearly 0%). Taken together, since the abrupt changes are fairly rare in the simulation settings, coverage appears correct (our method and others achieve approximately 95% coverage on average). But, ideally, (as the reviewer points out) public health would much prefer accurate coverage in periods when $\mathcal{R}_t$ is rapidly changing, precisely when these methods are the worst. [If $\mathcal{R}_t$ is flat near 1, then for our method, this is no different than if $\mathcal{R}_t$ is flat near 2: it will tend to over cover. But Bayesian methods with a prior that concentrates near 1 may completely fail to cover. The consequences for public health may be very different would be very different in these cases.] Furthermore, the shapes of the intervals are not believable: they should be asymmetric, getting wide below the estimates preceding a decrease in $\mathcal{R}_t$ (and the opposite for an increase). No method that we've seen produces these shapes. We would suggest that this is a major open problem in the field, and likely one without easy answers. We note that similar coverage issues plagued forecasting performance during the pandemic [16, 6].

Minor

Reviewer Point P 4.7 — p.2, l.4. See [9] for "instantaneous" vs. "case" effective reproduction number.

Reply: We've added the definition of instantaneous reproduction numbers in lines 6–8 in the manuscript, and we only focus on instantaneous reproduction number throughout the rest of the manuscript.

Reviewer Point P 4.8 — p.3, l.23 et seq. Bayesian approaches to learning Rt also enable any prior knowledge about the value of the parameter to be incorporated into inference. Some studies (e.g., [17]) have specified priors on Rt with prior mean substantially above 1 to ensure conservative posterior estimates.

Reply: Agreed. We've added discussion of these benefits in lines 60–62 in the manuscript.

Reviewer Point P 4.9 — p.3, l.51. This limitation, while definitely a valid point to make, does not necessarily apply to methods using conjugate priors (as mentioned by the authors on p.3, l.29), some of which can be computed almost instantaneously, so I would adjust the wording here.

Reply: Agreed. We've tried to be more careful here.

Reviewer Point P 4.10 — p.15. l.322. The derivation of the authors' divergence measure could be elaborated.

> **Reply**: We provided the derivation in Section A.1 in the supplementary document. We've also added text when it is first introduced, justifying our reasons for choosing this metric.

Reviewer Point P 4.11 — p. 22, l.436. Discussion of the importance and role of the k parameter, and best practices for setting it, could be slightly elaborated, with reference made back to some of the results earlier in the paper.

> **Reply**: This was a concern for many of the reviewers. We have expanded this discussion.

Reviewer 5

Minor

Reviewer Point P 5.1 — Line 102–104: the sentence seems to be missing a break between "... observed at discrete times (say, daily)" and "this delay distribution must be ...". Please rephrase if needed.

> **Reply**: Good catch. Thank you.

Reviewer Point P 5.2 — It's essential to choose the order k carefully for the smoothness of the estimator. Besides relying on domain knowledge and subjective preferences, are there more objective methods to determine the proper order? Are there any general suggestions for selecting k and assessing whether it's well-chosen?

> **Reply**: This was a concern for many of the reviewers. We have expanded this discussion in lines 398–407 in Section 3.1.2 in the manuscript.

Reviewer Point P 5.3 — In the simulations, why weren't all $k = 0, 1, 3$ evaluated in every scenario? While it's logical for k to correspond to certain R_t shapes, shouldn't we avoid basing the choice of k on synthetic R_t shapes, given that the true R_t wouldn't be known a priori in real-world data analysis? It would be insightful to assess the estimator's robustness by evaluating all $k =$ 0, 1, 3 and showcasing the estimates with these k values in all scenarios.

> **Reply**: This is a good point, and related to the previous point. We have adjusted the simulations as suggested.

Reviewer Point P 5.4 — Line 184, why the dimension of $D^{(1)}$ still contain k?, shouldn't it only contain n?

Reply: We should have written this more clearly. For all k, $D^{(k+1)}$ will have dimension $(n-k-1) \times n$. To create it using the iterative construction, the dimensions of $D^{(1)}$ have to be adjusted. We've added more explanation and adjusted the notation to clarify.

Reviewer Point P 5.5 — While performing cross-validation to select λ, are there any suggestions on determining the range of λ to search over?

Reply: We discussed this to some degree in Section 2.3, but we could have been more clear. One advantage of the ℓ_1 penalty is that there exists a value $\lambda_{\max}$ (the exact value, calculable at the outset, is given in Section 2.3), such that, for any $\lambda \geq \lambda_{\max}$, the estimate will be exactly the same. Therefore, there is no need to examine values larger than $\lambda_{\max}$. The software has a multiplier to pick $\lambda_{\min} = r\lambda_{\max}$ with the default $r = 10^{-4}$. In practice, we have not seen examples where the CV minimizer occurs at $\lambda_{\min}$, so there is no reason to consider smaller values. However, should this case occur, then one should simply adjust r and rerun the method until the CV minimizer is in the middle of the region. Alternatively, in the software, one can simply set $\lambda_{\min} = 0$ and examine the whole range at the outset. We have added some of this language to the discussion in lines 251–262 in Section 2.3 in the manuscript.

Reviewer Point P 5.6 — In line 433–434: For "…potentially advising public health to implement …", should it be something like "…potentially advising public health authorities to implement …"?

Reply: Yes, much better. Thank you.

References

[1] Abbott, S., Hellewell, J., Thompson, R. N., Sherratt, K., Gibbs, H. P., Bosse, N. I., Munday, J. D., Meakin, S., Doughty, E. L., Chun, J. Y. et al. (2020) Estimating the time-varying reproduction number of SARS-CoV-2 using national and subnational case counts. *Wellcome Open Research*, **5**, 112. `https://doi.org/10.12688/wellcomeopenres.16006.2`.

[2] Bergmeir, C., Hyndman, R. J. and Koo, B. (2018) A note on the validity of cross-validation for evaluating autoregressive time series prediction. *Computational Statistics & Data Analysis*, **120**, 70–83. URL: `https://www.sciencedirect.com/science/article/pii/S0167947317302384`.

[3] Boëlle, P.-Y., Ansart, S., Cori, A. and Valleron, A.-J. (2011) Transmission parameters of the a/h1n1 (2009) influenza virus pandemic: a review. *Influenza and other respiratory viruses*, **5**, 306–316.

[4] Bracher, J., Ray, E. L., Gneiting, T. and Reich, N. G. (2021) Evaluating epidemic forecasts in an interval format. *PLoS Computational Biology*, **17**, e1008618. URL: `http://dx.doi.org/10.1371/journal.pcbi.1008618`.

[5] Cori, A., Ferguson, N. M., Fraser, C. and Cauchemez, S. (2013) A new framework and software to estimate time-varying reproduction numbers during epidemics. *American Journal of Epidemiology*, **178**, 1505–1512. `https://doi.org/10.1093/aje/kwt133`.

[6] Cramer, E. Y., Ray, E. L., Lopez, V. K., Bracher, J., Brennen, A., Castro Rivadeneira, A. J., Gerding, A., Gneiting, T., House, K. H., Huang, Y. et al. (2022) Evaluation of individual and ensemble probabilistic forecasts of covid-19 mortality in the united states. *Proceedings of the National Academy of Sciences*, **119**, e2113561119.

[7] Ferguson, N. M., Cummings, D. A., Cauchemez, S., Fraser, C., Riley, S., Meeyai, A., Iamsirithaworn, S. and Burke, D. S. (2005) Strategies for containing an emerging influenza pandemic in southeast asia. *Nature*, **437**, 209–214.

[8] Genovese, C. and Wasserman, L. (2008) Adaptive confidence bands. *The Annals of Statistics*, **36**, 875 – 905. URL: `https://doi.org/10.1214/07-AOS500`.

[9] Gostic, K. M., McGough, L., Baskerville, E. B., Abbott, S., Joshi, K., Tedijanto, C., Kahn, R., Niehus, R., Hay, J. A., De Salazar, P. M. et al. (2020) Practical considerations for measuring the effective reproductive number, R_t. *PLoS Computational Biology*, **16**, e1008409. `https://doi.org/10.1371/journal.pcbi.1008409`.

[10] Gressani, O., Wallinga, J., Althaus, C. L., Hens, N. and Faes, C. (2022) Epilps: A fast and flexible Bayesian tool for estimation of the time-varying reproduction number. *PLoS Computational Biology*, **18**, e1010618. `https://doi.org/10.1371/journal.pcbi.1010618`.

[11] Groendyke, C., Welch, D. and Hunter, D. R. (2011) Bayesian inference for contact networks given epidemic data. *Scandinavian Journal of Statistics*, **38**, 600–616.

[12] Lipsitch, M., Cohen, T., Cooper, B., Robins, J. M., Ma, S., James, L., Gopalakrishna, G., Chew, S. K., Tan, C. C., Samore, M. H. et al. (2003) Transmission dynamics and control of severe acute respiratory syndrome. *science*, **300**, 1966–1970.

[13] Parag, K. V. (2021) Improved estimation of time-varying reproduction numbers at low case incidence and between epidemic waves. *PLoS Computational Biology*, **17**, e1009347. `https://doi.org/10.1371/journal.pcbi.1009347`.

[14] Park, S. W., Akhmetzhanov, A. R., Charniga, K., Cori, A., Davies, N. G., Dushoff, J., Funk, S., Gostic, K., Grenfell, B., Linton, N. et al. (2024) Estimating epidemiological delay distributions for infectious diseases. *medRxiv*, 2024–01.

[15] Park, S. W., Sun, K., Champredon, D., Li, M., Bolker, B. M., Earn, D. J., Weitz, J. S., Grenfell, B. T. and Dushoff, J. (2021) Forward-looking serial intervals correctly link epidemic growth to reproduction numbers. *Proceedings of the National Academy of Sciences*, **118**, e2011548118.

[16] Rosenfeld, R. and Tibshirani, R. J. (2021) Epidemic tracking and forecasting: Lessons learned from a tumultuous year. *Proceedings of the National Academy of Sciences*, **118**, e2111456118.

[17] Thompson, R. N., Stockwin, J. E., van Gaalen, R. D., Polonsky, J. A., Kamvar, Z. N., Demarsh, P. A., Dahlqwist, E., Li, S., Miguel, E., Jombart, T. et al. (2019) Improved inference of time-varying reproduction numbers during infectious disease outbreaks. *Epidemics*, **29**, 100356.
