# Supplementary material for: rtestim: Time-varying reproduction number estimation with trend filtering
Source: PLoS Comput Biol. 2024 Aug 6;20(8):e1012324. doi: 10.1371/journal.pcbi.1012324 (PMC11329163; doi:10.1371/journal.pcbi.1012324)
Supplement: Attachment — Submitted filename: PLoS-second-responce.pdf [file pcbi.1012324.s005.pdf]

Response to the reviewers

We appreciate all reviewers for their positive feedback and thank the reviewer for bringing up the inconsistency of the method name and software name.

Reviewer 2's comment

One small suggestion is that there should perhaps be a consistency of naming of the package: it is interchangeably referred as both RtEstim and rtestim.

> **Reply**: We now stick with `rtestim` to refer to both of the method and software throughout the manuscript.
