# Supplementary material for: rtestim: Time-varying reproduction number estimation with trend filtering
Source: PLoS Comput Biol. 2024 Aug 6;20(8):e1012324. doi: 10.1371/journal.pcbi.1012324 (PMC11329163; doi:10.1371/journal.pcbi.1012324)
Supplement: Attachment — Submitted filename: PlosCB review Feb 2024.docx [file pcbi.1012324.s003.docx]

RtEstim: Effective reproduction number estimation with trend Filtering

The authors present a new frequentist approach to estimating the instantaneous reproductive number using the renewal equations. Their modifications allow for a more sophisticated smoothing process and show good results in simulations. However, the rationale for the simulation scenarios that are selected is not clear and should be better justified.

1. (minor) l2-3. The instantaneous reproductive number is a type of effective reproductive number. These are not interchangeable.
2. There should be more citations in the first paragraph of the introduction.
3. It is unclear why the serial interval parameterizations are being changed for each simulation scenario and how this impacts the results. If these are shape and scale parameters, then this would imply really large means and variances (e.g. mean=3.5*3.5=12.25 and variance=3.5^3). It is not clear how these distributions are motivated.
4. The negative binomial simulations chose a dispersion parameter that is relatively large and it is not clear that is a really strong violation of the Poisson assumption (unless some other parametrization of the distribution is being used).
5. It would be helpful to see the method compared to EpiFilter in a setting where the case counts are smaller and EpiFilter would work. These scenarios are frequently of interest.
6. The choice of k varies for each scenario-how is one to decide what to use?
7. It would be helpful to see how sensitive the simulation results are to the assumed serial interval distribution. Presumably some methods should be more sensitive to this than others.
8. It would be helpful to compare RtEstim with EpiEstim and other approaches for the real-world examples, too. This would provide insight on whether the patterns observed in the simulations hold up with real data.
9. I do not really follow the discussion on the reporting delay being inconsequential.
