# Supplementary material for: rtestim: Time-varying reproduction number estimation with trend filtering
Source: PLoS Comput Biol. 2024 Aug 6;20(8):e1012324. doi: 10.1371/journal.pcbi.1012324 (PMC11329163; doi:10.1371/journal.pcbi.1012324)
Supplement: Attachment — Submitted filename: Referee report -- Liu 2024 (1).docx [file pcbi.1012324.s002.docx]

In “RtEstim: Effective reproduction number estimation with trend filtering” Liu et al. develop a novel estimator for Rt. Based on discrete splines, the approach can accurately model rapid changes in Rt while still producing smooth estimates via an automated adaptive smoothing procedure. The frequentist estimation procedure is also extremely efficient, much quicker than leading Bayesian approaches. The manuscript is well-written and informative, with a thorough survey of the the many new Bayesian and frequentist approaches that have been developed in the last few years.

However, I believe there are some important additional validations necessary. In particular, I would like to know more about how the method performs under generation interval (GI) misspecification. I also had some additional questions around the metrics used in evaluation and the models being compared. I split my questions in major and minor concerns, listed below:

**Major**

- Before I felt comfortable using this estimator, I would want to see additional analyses examining robustness to GI misspecification. Is this automated smoothing procedure robust to misspecified GIs? Could it potentially compound GI misspecification, adding additional bias? On the other hand, it seems possible that the smoothing induced by the penalty might could reduce the bias from GI misspecification. I’d be curious to see the impact on the simulated situations in Figure 2.
- Do the confidence intervals cover Rt at the nominal levels? Is this true in the negative binomial case? What about for predicted incident cases?
- In lines 65-66, the authors describe this estimator as a “retrospective effective reproductive number”. I’m confused by this phrase. Does it mean that the proposed method is not expected to forecast well and should only be used retrospectively? Or does it mean that the estimator is a case reproductive number rather than an instantaneous reproductive number [1]? If it is a case reproductive number, differences with the Cori method’s instantaneous reproductive number should be discussed.
- For the comparisons, why use EpiEstim vs. the newer and more robust EpiNow2? The GP in EpiNow2 also provides smoothing, so it should be a stronger comparison.
- I’m not sure the timeseries of Rt in Figures 1 & 7 are appropriate because of the change in the GI during the several years displayed. I would think the bias would be particularly severe during the Omicron wave, when the model is at its most certain. It would probably be more appropriate to use different GIs for before and after Omicron’s emergence (with different model fits perhaps).

**Minor**

- I had some trouble following the notation in line 184. Does the D^1 case correspond to k = 0? If so, it would be helpful to say that explicitly.
- In Figures 4 & 5, I had trouble distinguishing the colors in all the different situations.
- In Figures 4 & 5, does the RtEstim (k=0) case appear in all the panels?
- I don’t believe the norm used in section 2.3 is ever defined. Is it the L1 norm?
- I would appreciate seeing the values plotted in Figures 4 & 5 as the difference between the fitted and true values, perhaps as additional supplementary figures. It’s nice to see the timeseries, but I have trouble reading the figures.
- In Section 2.4, I would appreciate some discussion of why random splitting into k folds is more appropriate than leave future out validation.
- I don’t think the claims on line 430-440 in the Discussion around epidemic forecasting and real-time usage are appropriate or substantiated by the analysis. The authors do not evaluate performance at forecasting Rt and, without seeing an evaluation, I am skeptical that splines would outperform other methods.
- I would appreciate some more discussion of outlier handling. In several places, it is mentioned that outliers are excluded but I couldn’t find what qualified as an outlier or why.
- When discussing the generation interval, its approximation by the serial interval, and discretization procedures it would be good to discuss the results in [2] and [3].

[1] Gostic, Katelyn M., et al. "Practical considerations for measuring the effective reproductive number, R t." *PLoS computational biology* 16.12 (2020): e1008409.

[2] Park, Sang Woo, et al. "Forward-looking serial intervals correctly link epidemic growth to reproduction numbers." *Proceedings of the National Academy of Sciences* 118.2 (2021): e2011548118.

[3] Park, Sang Woo, et al. "Estimating epidemiological delay distributions for infectious diseases." *medRxiv* (2024): 2024-01.
